# The morning after: what now for psychiatry research?

**DOI:** 10.1186/s12916-016-0655-x

**Published:** 2016-07-15

**Authors:** Simon Wessely, Krista Nicholson

**Affiliations:** Department of Psychological Medicine, Institute of Psychiatry, Psychology and Neurosciences, Kings College London, Weston Education Centre, Cutcombe Road, London, UK; Royal College of Psychiatrists, 21 Prescot Street, London, UK

## Abstract

The UK scientific community is rightly concerned about the impact of leaving the EU on UK science. These concerns are particularly pertinent for mental health research, which is chronically underfunded in comparison to research on physical health conditions. The EU is one of the largest funders of mental health research in the world, with the UK clearly benefitting from this because of its strong track record. Any loss of funding, leadership or influence would weaken this. Likewise if we are unable to attract the best or most promising researchers from the rest of Europe, the loser will not just be research into mental health across Europe, but patients themselves. Those working on the Brexit negotiations must develop clear and coherent plans to safeguard scientific research in UK and ensure that the momentum gained in mental health policy in recent years is not lost.

## Editorial

The future of UK science was not much of an issue during the EU referendum campaign. This was probably because most of the voters don’t think about science most of the time. Furthermore, the Leave campaign, which seemed to lead the debate, would have been foolish to push science up the agenda. The UK’s science community, including all those in health and medicine, overwhelmingly supported remaining in the EU. The British Medical Journal was unable to “*name one prominent national medical, research, or health organization that has sided with Brexit*” [1].

It is not hard to understand why. The UK has hugely benefited from EU research funding, receiving €8.8 billion between 2007-2013 despite only contributing €5.4 billion to the EU research budget over the same period [2]. Under the current Horizon 2020 research programme the UK has won more projects than any other country [3], and also leads on more collaborations than any other country. Expressions of support to remain came from 5,500 UK scientists [4], all the ex-Presidents of the Academy of Medical Sciences [5], 71 ex-Presidents of the Medical Royal Colleges [6], 13 Nobel Laureates [7] and 150 Fellows of the Royal Society [8] and so on. If the vote had been decided on expertise, it would have been very one sided. But in what may turn out to be Michael Gove’s only contribution to dictionaries of quotations, the public seemed to have “had enough of experts”.

And now we have to deal with the consequences. Sometimes after contested, emotion driven elections, even when things don’t go the way one hoped, it doesn’t seem so bad when the noise has died down. Not so for science. Professor Sir Robert Lechler, President of the Academy of Medical Sciences, has called the referendum result “*the most serious threat and challenge to UK research in my lifetime*” [9].

We are particularly concerned about the implications for mental health research, which dispiritingly remains chronically underfunded in comparison to research on physical health conditions, despite similar returns on investment. In the UK we invest less than 6 % of the annual health research budget on mental illness (£115 million), [10] despite its accounting for around 23 % of the disease burden [11]. In a sector where every penny counts, UK researchers cannot afford to miss out on EU support.

The EU remains the largest single funder of mental health research in Europe and indeed one of the 10 largest funders globally [12]. FP7, for example, invested €1.92 billon in brain research between 2007 and 2012, [13]. But it’s not just about money. Like all science, research into psychiatry/neuroscience is a collaborative enterprise which benefits from people working together and sharing ideas and resources, and the EU is at the heart of that. At present there are several major collaborations led by UK scientists. NEWMEDS is a major initiative linking eight universities and ten pharm/biotech companies addressing schizophrenia and depression [14]. Then we have EU AIMS (autism treatment) [15], EU MATRICS (aggression) [16], EU TACTICS (obsessive compulsive disorder) [17], EU BRAINVIEW (training young scientists in brain development) [18], MILESTONES (improving transition between child and adult mental health series) [19] and RADAR CNS, funded from IMI2, linking universities, five pharmaceutical and two software companies, testing the utility of digital phenotyping to assess clinical state and predict relapse in multiple sclerosis, epilepsy and recurrent depression [20].

The importance of collaboration for the future of mental health research across Europe was confirmed by the 2015 ROAMER (“Road Map for Mental Health Research in Europe”) project, the largest ever exercise for setting out ‘*a comprehensive, coordinated mental health research agenda for Europe*’ and again UK led [21]. The ROAMER recommendations have also been incorporated into the NHS-England Five Year Forward View for Mental Health [22].

With this in mind, there could not be a worse time for UK researchers to be marginalised from mental health research in the rest of Europe, and vice versa. The UK is a powerhouse of mental health research, undertaking more studies than any other country in Europe, with its largest institution, the Institute of Psychiatry, Psychology and Neuroscience, ranked first in the world according to Thomson’s Essential Science Indicators. No one who cares about mental health and neuroscience research, on both sides of the Channel, will want to see UK science isolated.

Science benefits from easy movement of labour. EU freedom of movement regulations, which allow researchers and clinicians to work in any member state, are one reason that around one in six (16 %) of all academic staff at UK Universities are non-UK EU nationals [23]. And it would be hard to think of a UK research project that had not benefited from the contributions of interns, PhD students or research fellows from across the region.

But perhaps the biggest impact is the hardest to quantify. This is the potential negative impact on our ability to attract high quality researchers. As the House of Lords Science and Technology Select Committee warned before the vote, “*researcher mobility must be protected if UK science and research is to remain world-leading*” [24].

So we currently face years of uncertainty around the rights of EU nationals already in the UK and for those looking to come here in the future, as well as uncertainty around UK access to the ERASMUS scheme and around the amount of fees EU students will pay to study at UK universities. This is much more than simply the right to remain; it involves all the benefits that come with EU citizenship, including recognition of qualifications, portability of pensions, access to health care and much else. Anecdotal evidence is already accumulating that researchers are being put off accepting jobs in the UK. We need swift action to reassure that we are “open for business”. 

Likewise, we remain in the EU for at least two more years, during which time UK scientists are still eligible to apply under all EU science schemes, and any grants awarded will be honoured for the duration of the award. There is already confusion about this and evidence that UK scientists are already being dropped from EU science projects because of funding fears [25]. The grass roots science campaign, “Scientists for the EU” are currently collecting data on all these issues to inform policy, negotiations and that existing rights remain honoured – there is a website for recording these issues as soon as they arise –(http://scientistsforeu.uk/monitoring-brexit-impact/).

One outcome would be that the Brexit negotiations result in the UK achieving “associated country” status, similar to the Norwegian model, which would allow us to “buy into” the EU science programme and apply for Horizon 2020 projects in the same way as if we had remained an EU member state. However, associated country status is not guaranteed and a pre-condition for participation will almost certainly be continued freedom of movement between EU countries, which almost certainly seems unpalatable to those who support Leave. If politicians decide the referendum is a mandate to limit migration, we could suffer the same fate as Switzerland following their 2014 referendum vote to limit mass migration. Initially suspended from Horizon 2020, they have now been relegated to a “partially associated” status and forced to increase national spending to try and match the funds lost. By some estimates this has reduced their participation in EU research projects by 40 % [26].

Associated country status is no panacea and would be a serious blow to the UK’s reputation as a heavyweight in European science. We would no longer be able to participate in negotiations over any future EU funding strategies (which we have previously played a huge role in shaping) [27] and we would also lose influence over other EU policies, (such as the Clinical Trials and Data Protection directives, both of which ended well, but only because of our active lobbying and influence), which we would either be forced to adopt or again risk remaining out in the cold.

Despite this gloomy outlook, with the referendum behind us, there is a now a clear agenda for all those who believe science plays a vital role in improving our health and wellbeing. Those of us who supported Remain must do everything we can not to be proven right in the fears we expressed during the campaign. Those who supported Leave likewise now need to be clear how they will make good on their promises for the NHS, and come up with some clear and coherent plans to safeguard scientific research, and do so without delay. We must ensure that mental health is not forgotten in these negotiations, and that the momentum of previous years of work is not lost. Mental health research funding needs a significant uplift and the UK must remain a key player in pushing forward the ROAMER agenda in Europe.

One of the main leaders of the Leave campaign had a good line in Shakespearean and classical allusions. So continuing the theme, we note there is a tide in the affairs of men, which if we don’t get right, means that researchers, mental health professionals and patients across Europe will wallow in the shallows while others are led on the current to fortune.
